# A Novel Passive Method for the Assessment of Skin-Electrode Contact Impedance in Intraoperative Neurophysiological Monitoring Systems

**DOI:** 10.1038/s41598-020-59551-w

**Published:** 2020-02-18

**Authors:** Eduardo Alonso, Romano Giannetti, Carlos Rodríguez-Morcillo, Javier Matanza, José Daniel Muñoz-Frías

**Affiliations:** Institute for Research in Technology, ICAI School of Engineering, Pontifical Comillas University, Madrid, Spain

**Keywords:** Health care, Biomedical engineering

## Abstract

Intraoperative Neurophysiological Monitoring is a set of monitoring techniques consisting of reading electrical activity generated by the nervous system structures during surgeries. In order to guarantee signal quality, contact impedance between the sensing electrodes and the patient’s skin needs to be as low as possible. Hence, monitoring this impedance while signals are measured is an important feature of current medical devices. The most commonly used technique involves injection of a known current and measurement of the voltage drop in the contact interface. This method poses several problems, such as power consumption (critical in battery-powered systems), frequency dependency and regulation issues, which are overcome by using a passive method. The fundamentals of the method proposed in this paper are based on the utilization of the variation suffered by the input random signal when a known resistance is connected in parallel to the input terminals of the low-noise amplifier (LNA) of the analog front-end of the acquisition system. Controlling the connection of the resistors and computing the root mean square of the LNA output voltage has been proved to be a useful tool to assess that the contact impedance is suitably low, allowing the user to know if the neural measurements obtained are valid.

## Introduction

Intraoperative Neurophysiological Monitoring (IONM) is a set of techniques that provide increased functional knowledge of the nervous system structures during a surgical operation. These powerful tools allow the surgeon to identify and prevent possible injuries during the medical procedure^1^. The IONM denomination includes stimulus-based techniques, such as evoked potentials (that measure the response to a previous stimulation), and free running techniques, which can be acquired continuously without any stimulus, including Electroencephalography (EEG), Electromyography (EMG) and Electrocorticography (ECoG), and several other.

Due to the context of utilization, it is of paramount importance to guarantee the accuracy of the biological signals acquired. Therefore, limiting the noise sources and their effects over the measurements is a critical aspect of IONM.

**Figure 1:**
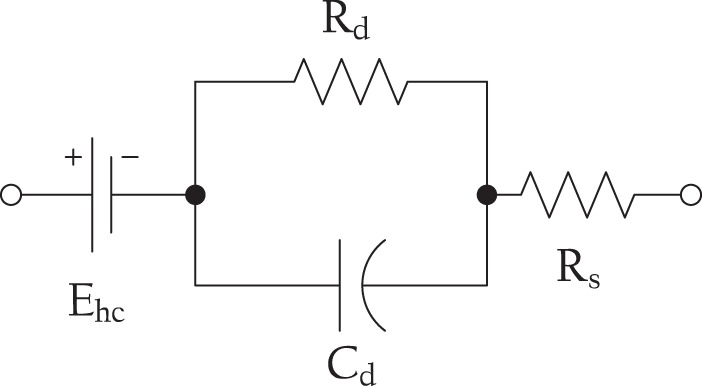
Equivalent circuit for a biopotential electrode-tissue impedance. ${E}_{hc}$ is the half-cell potential, ${R}_{d}$ and ${C}_{d}$ the impedance due to the electrode-electrolyte and polarization effects, and ${R}_{s}$ models the interface effects due to the resistance of the electrolyte^3^.

A common source of signal quality degradation is the contact impedance that appears in the interface between the electrode and the patient’s tissues^2^. Commonly, IONM techniques use needle electrodes and/or wet adhesive electrodes for the connections, creating an interface between the metal end of the electrode and the biological tissue. The electrical behaviour of this interface is normally represented following the Webster model^3^, as can be seen in Fig. 1.

A large contact impedance may lead to a system more susceptible to power line interferences and motion artefacts^4,5^; additionally, the voltage drop on the same impedance results in signal attenuation^6^. Kappenman and Luck^7^ highlight the importance of electrode impedance on data quality for event-related potentials (ERP) and EEG signals. Sagha *et al*.^8^ include high electrode impedance as a source of signal degradation for EEG utilized in brain-computer interfaces. In order to avoid these pernicious effects, the literature sets the limit for the impedance value around $5$ kΩ for EEG^6,9–12^ and for evoked potentials^1^.

Typically, the first stage of a biopotential monitoring system consists of a low-noise amplifier (LNA) that receives the analog signal sensed by several electrodes connected to the patient. A high-impedance first stage is necessary for a system of this kind^13^, because it minimizes the effect of the contact impedance. Nevertheless, and due to the half-cell potential that occurs in the interface between the electrode and the biological tissues (${E}_{hc}$ in Fig. 1), it is necessary to remove the DC component to prevent the signal from saturating the output of the amplifier. The design of a passive high pass filter (HPF) with a low cutoff frequency requires the use of a parallel resistor that lowers the input impedance of the system in several orders of magnitude with respect to the input impedance of the LNA.

The common method for measuring the electrode-tissue impedance consists of injecting a known AC current^14^ (usually a square wave) and measuring the differential voltage between the electrodes^6,15^; this methodology is applied to measure bio-impedance in general. For example, Zamani *et al*.^16^ present a bio-impedance measurement system for cardiac tissues, and Rodriguez *et al*.^17^ describe a wireless sensor suitable for implantable devices. Multifrequency analysis can be found in the literature, applied for example to cardiac tissues^18^, in order to extend the analysis of the impedance to the range of frequencies of the measured signals. Kubendran *et al*.^19^ highlight the drawbacks of using sinusoidal sources or square waves when performing an analysis of this kind.

Current injection presents several problems. Firstly, the application of an active current into the patient is restricted due to regulation of medical devices, implying a thorough validation process for Class B or Class C devices (those that may cause a potential injury to the patient)^20^. The devices that do not inject current are Class A devices, and their validation process is easier. A second drawback of this method is power consumption, due to the utilization of current generators and dedicated circuitry to measure the skin-electrode impedance. The devices that do not inject current to the patient do not need additional current generators, and so they are going to consume less power. This issue is especially relevant given that recent years have witnessed the proliferation of wireless designs for IONM^21,22^, which could mark the beginning of battery-powered systems. An additional issue is the abrasion of the skin when measurements are constant and repeated, needed to maintain the signal quality, which requires tedious scalp conditioning that may lead to an increase of infection risk^6^ and of skin diseases; being able to monitor the contact impedance often without the need of injecting any current in the tissue will permit to extend the time between electrode repositioning or skin treatments.

On the other hand, the exact value of the impedance is often not interesting for the IONM application; the only information required is an almost binary flag indicating if the connection is *good enough* for a correct measurement, or if the placement of the electrode must be corrected. Several instruments just report if the value is safely lower, more or less around, or clearly higher than a designated threshold–which is typically near 5 kΩ.

This paper presents a novel impedance measurement method that overcomes the problems presented by the injection of current (power consumption, different class of the device for the validation process, skin abrasion) by evaluating the effect of a change in the input resistance of the LNA on the measured signal. A description of the methodology proposed is presented in Section 2. Section 3 shows the experimental arrangement carried out to validate the system and the results obtained. Conclusions of the study are stated in Section 4.

Most of the experiments in our paper were carried out using a prototype instrument, with an “ad-hoc” testing system developed to simulate the EEG signals in shape, value and impedance, and using a synthesized signal. In the last part of Section 3, preliminary results of a new measurement and test campaign on volunteer human subjects is also reported.

## Proposed Methodology

**Figure 2:**
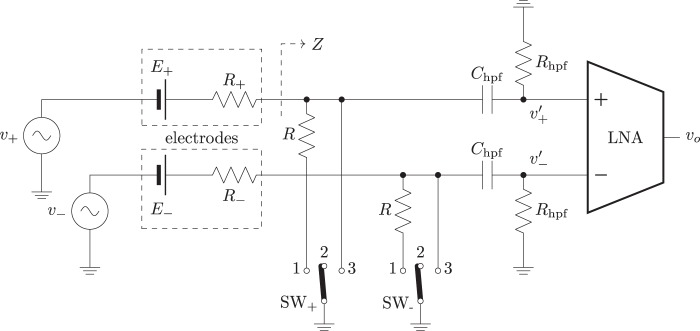
Simplified schematic of system implementing the measurement technique.

The passive method (without the injection of a probe current) proposed here is based in the changes in the measured signal when a purely resistive load is connected in parallel to either the positive or the negative input terminals of the LNA. Altough similar techniques are found in the literature^6^, in this proposal the signal analyzed to assess the impedance is the biological input signal and not a signal known *a priori*. The hardware implementation for the system is depicted in Fig. 2.

In this work, the contact between electrode and skin is modeled as a series resistor and a DC voltage source. This simplification with respect to the Webster model^3^ presented in Fig. 1 is made possible thanks to the following considerations:

- Firstly, the tissue impedance term (${R}_{s}$ in Fig. 1) is neglected since it is generally at least two orders of magnitude smaller than the contact impedance^2^.
- Secondly, measuring the value of the reactive elements is important for the frequency-domain analysis of the contact impedance; but in this method the effect of this impedance is assessed with the same signal which is being measured, so that we can consider the contact resistance as an averaged-out equivalent in the band of interest.
- Finally, the intent of the measurement is to just estimate if the contact impedance is *low enough* for a correct measurement, and not a value for the impedance itself.

The half-cell potential due to the interface between each electrode and the tissue in the contact interface is modeled, as shown in Fig. 2, by two batteries ${E}_{+}$ and ${E}_{-}$. These potentials will be similar in all the terminals due to the utilization of the same type of electrodes. The effect of these DC voltage sources is filtered out by the high pass filter at the input terminals of the LNA, composed by the capacitors ${C}_{hpf}$ and resistors ${R}_{hpf}$. ${R}_{+}$ and ${R}_{-}$ model the resistive behaviour of the contact between the electrode and the skin of the patient for each electrode. Finally, the signal to be measured is modeled by the pair of generators ${v}_{+}$ and ${v}_{-}$ that represent the ideal (unknown) pair of monopolar biopotentials that is to be measured.

If the amplifier is considered ideal, the output of the circuit will follow

1 $${v}_{o}=G\cdot ({v{\prime} }_{+}-{v{\prime} }_{-})$$

where $G$ is the gain of the LNA and ${v{\prime} }_{+}$ and ${v{\prime} }_{-}$ are the voltages at each input terminal of the amplifier in the frequency domain.

The position of switches $S{W}_{+}$ and $S{W}_{-}$ determine the relationship between ${v{\prime} }_{+}$ and ${v}_{+}$ and, in the same way, between ${v{\prime} }_{-}$ and ${v}_{-}$. Position $1$ connects a resistor of known value ($R$) in parallel to the input, while position $3$ grounds the respective input, setting it to zero. When the switches are in position $2$, the system is in normal operation.

In normal operation mode, the voltage at the input of the positive terminal of the LNA can be calculated, ignoring the effect of the DC voltage source due to the filter and assuming that the input impedance of the amplifier is large enough:

2 $${v{\prime} }_{+}={v}_{+}\cdot \frac{{R}_{hpf}}{{R}_{hpf}+\frac{1}{j\omega \cdot {C}_{hpf}}+{R}_{+}}$$

Assuming that the frequency of the signal and the value of the resistor of the filter are large enough with respect to the value of the contact impedance, (2) can be simplified:

3 $${v{\prime} }_{+}\simeq {v}_{+}$$

The same calculation is carried out when the parallel resistor is connected to the positive input of the LNA ($S{W}_{+}$ in position 1 in Fig. 2):

4 $${v{\prime} }_{+}={v}_{+}\cdot \frac{Z}{{R}_{+}+Z}\cdot \frac{{R}_{hpf}}{{R}_{hpf}+\frac{1}{j\omega \cdot {C}_{hpf}}}$$

where $Z$ can be calculated as

5 $$Z=\frac{({R}_{hpf}+\frac{1}{j\omega \cdot {C}_{hpf}})\cdot R}{{R}_{hpf}+\frac{1}{j\omega \cdot {C}_{hpf}}+R}$$

Assuming again that the frequency of the signal is large enough with respect to the cutoff frequency of the filter, and considering that the values of $R$ and ${R}_{hpf}$ are known and ${R}_{hpf}\gg R$, (5) can be simplified to

6 $$Z\simeq \frac{{R}_{hpf}\cdot R}{{R}_{hpf}+R}\simeq R$$

and therefore, (4) becomes

7 $${v{\prime} }_{+}={v}_{+}\cdot \frac{R}{{R}_{+}+R}$$

that corresponds to the equation of a resistive voltage divider, similar to what is calculated in by Ferree *et al*.^6^. The same procedure can be applied to the negative terminal input of the system.

Consequently, a difference in the input signals measured is observed when the parallel resistor $R$ is connected to any of the electrodes. One can define $\alpha$ and $\beta$ as the attenuation coefficients caused by the relation between the contact impedance ${R}_{+}$ and ${R}_{-}$ and the known resistance ($R$) when the corresponding switches are closed.

8 $$\alpha =\frac{R}{R+{R}_{+}}\,\,\beta =\frac{R}{R+{R}_{-}}$$

These coefficients become useful when defining a set of voltage measurements that can be obtained when combining the different positions of the switches shown in Fig. 2:

9 $$\begin{array}{llll}{v}_{a} & = & G\cdot ({v}_{+}) & \mathrm{(2,3)}\\ {v}_{b} & = & G\cdot (\alpha \cdot {v}_{+}) & \mathrm{(1,3)}\\ {v}_{c} & = & G\cdot (\,-\,{v}_{-}) & \mathrm{(3,2)}\\ {v}_{d} & = & G\cdot (\,-\,\beta \cdot {v}_{-}) & \mathrm{(3,1)}\end{array}$$

where the position of $S{W}_{+}$ and $S{W}_{-}$ are indicated, in this order, between brackets.

To apply the method for the assessment of the impedances, the voltages in (9) are measured consecutively. More specifically, ${v}_{a}$ is obtained when the negative terminal is connected to the system ground ($S{W}_{-}$ in position 3) and the positive terminal is in normal operation mode ($S{W}_{+}$ in position 2). Subsequently, $R$ is connected in parallel to the positive terminal ($S{W}_{+}$ in position 1) and ${v}_{b}$ is measured. Then ${v}_{c}$ and ${v}_{d}$ are measured in a similar way, with the positive terminal grounded ($S{W}_{+}$ in position 3) and $S{W}_{-}$ toggled between positions $2$ and $1$, respectively.

Thus, $\alpha$ and $\beta$ can be calculated independently by means of the measurements taken, following

10 $$\alpha =\frac{{v}_{b}}{{v}_{a}}\,\,\beta =\frac{{v}_{d}}{{v}_{c}}$$

In the general case, these voltages are variable in time, and from the perspective of the method, they can be considered almost as they were random, or random-like signals. To obtain a value for $\alpha$ and $\beta$, which are needed to assess the values of ${R}_{+}$ and ${R}_{-}$, a statistical value extracted by the measurements is applied to the equations in 9, such as the Root Mean Square (RMS) value. Calling *F*(·) the function that calculated the designated statistical value from each voltage, we have:

11 $$\alpha =\frac{F({v}_{b})}{F({v}_{a})}\,\,\beta =\frac{F({v}_{d})}{F({v}_{c})}$$

Finally, with the help of 8, the skin-electrode contact impedance can be calculated from the measurements taken:

12 $${R}^{+}=R\cdot \frac{1-\alpha }{\alpha }\,\,{R}^{-}=R\cdot \frac{1-\beta }{\beta }$$

## Experimental Setup

In order to verify the system, firstly the method has been evaluated mathematically (using ideal, simplified models for the components) and by simulation with the full models of the electronic circuit. Afterward, a laboratory test system that simulates the signal sources has been implemented, and the method has been validated using synthetic signals recorded by real EEG and ECoG. Finally, a preliminary test was carried out with a prototype on human volunteers, with satisfactory results.

### Mathematical model and simulations

**Figure 3:**
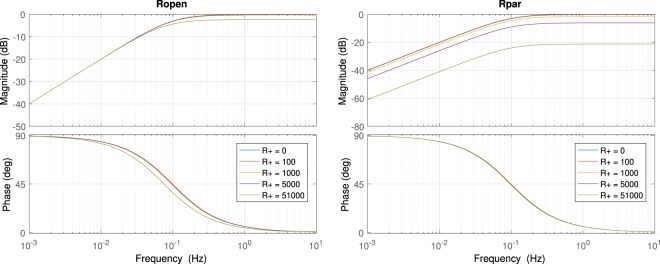
At the left, Bode diagram of ${v{\prime} }_{+}/{v}_{+}$ with ${v{\prime} }_{-}$ grounded, in normal operation mode; at the right, with a 5 kΩ parallel resistor connected, for several contact resistance values (values of ${R}_{+}$ in Ω).

As a first step, we checked that the effects of the added resistors $R$ on the frequency response of the system are acceptable. Two different configurations have been studied: in normal operation mode and when the parallel resistor $R$ is placed in the positive input. The Bode diagrams of the frequency response of the system, for several values of the contact resistor ${R}^{+}$, are shown in Fig. 3; the first one represents the transfer function ${v{\prime} }_{+}/{v}_{+}$ in normal operation, and the second one shows the same transfer function when a resistor $R=5{\rm{k}}\varOmega$ is connected (SW + in position 1).

As can be seen from the figures, the frequency response of the system is almost constant when the contact impedance changes and no resistor is placed in parallel. On the other hand, when the parallel resistor is connected, an increase in the value of the contact resistor leads to a signal attenuation basically constant in the frequencies of interest. Nevertheless, the cut-off frequency of the input high-pass filter is not altered in any scenario.

To test the method, we calculated the output voltage for a sinusoidal voltage with a frequency of 1 kHz as input. The output voltage of the signal is analyzed and the impedance estimated with the help of equations (9), (11) and (12), and then compared with the real value.

The root mean square (RMS) value of the input signal is selected as the statistical property (function *F*(·) in Eq. 11), because it offers a good trade-off between robustness and computational cost.

Three different cases are possible, depending on the values of the contact resistances of the two inputs. Firstly, if the contact impedance is much lower than 5 kΩ, the voltage drop in ${R}_{+}$ (or ${R}_{-}$) due to the connection of $R$ will be negligible and the coefficients $\alpha$ (or $\beta$) will be close to $1$. Secondly, a contact impedance around 5 kΩ will give values for $\alpha$ or $\beta$ close to $0.5$. Finally, in the case that ${R}_{+}$ is much greater than $R$, the voltage drop in the contact impedance will be noticeable, and hence the coefficients will reach a value close to $0$.

**Figure 4:**
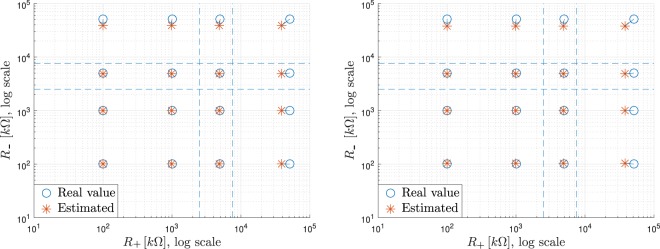
On the left, result of the calculations performed on the simplified model; on the right, result of the simulations with the full circuit model. Value of resistive impedances estimated vs. calculated.

The results of this estimation are shown in the left diagram in Fig. 4, where the calculated impedance is represented in comparison to the real values of the resistors tested. A total of sixteen cases have been represented, resulting from the combination of the values 100 Ω, 1 kΩ, 5 kΩ and 51 kΩ for ${R}_{+}$ and ${R}_{-}$. This set of values has been chosen because the border between acceptable and non-acceptable contact impedance has been set at 5 kΩ, and to explore the behavior with similar or very different values. In the result, the differences arise basically from rounding errors in the operations, mainly due to the numerical calculation of RMS.

Since the aim of this methodology is the assessment of the contact impedance between electrode and skin, two thresholds are arranged in order to classify the impedance as good, middling or unacceptable. In the figure, these two thresholds are set to the values of $R=2.5\,{\rm{k}}\Omega$ and 7.5 kΩ, providing a separation between nine different scenarios, each one identified by the combination of one of the three possible classes of the real contact impedance and one of the three possible outcomes of the measurement. In other words, the objective of the method is that real and measured impedances share the same scenario, meaning that a good contact impedance is estimated as good, a middling as middling, and so on.

As expected, and as can be seen from Fig. 4, the impedances are correctly estimated in all scenarios, when the model is evaluated mathematically.

Once the methodology has been validated through a mathematical model where the components are represented in ideal conditions, a set of simulations have been carried out in order to confirm the validity of this method taking into account the full model of the active components of the circuit.

The freely available LTSpice simulation software has been utilized to simulate the corresponding circuit. The AD8429 low noise amplifier, which is the one chosen for the prototype, has been simulated using the model made available by the manufacturer. The switches that connect the resistor $R$ in parallel to the inputs are modeled with a voltage-controlled switch, so that the four configurations stated in (9) are simulated sequentially.

A sinusoidal voltage at 1 kHz with a peak to peak value of 15 μv and an offset value of 1 mV is utilized as input. Each configuration switching is followed by a transient period due to the response of the HPF located in both terminal inputs of the LNA. Due to the transient, the RMS value has to be computed after the signal is settled. Considering a trade-off between the duration of the algorithm and the precision, the calculation of the RMS is carried out after $2\tau$.

Again, sixteen cases are simulated, combining four impedance values (100 kΩ, 1 kΩ, 5 kΩ and 51 kΩ) for both ${R}_{+}$ and ${R}_{-}$. The comparison between the calculated and the real impedance is represented in the right diagram in Fig. 4. In this figure it can be seen that, even though the estimation of the impedances is fairly good, the estimation error has increased slightly with respect to the theoretical analysis. The identification of the working scenario of the system with respect to the impedance condition (good, middling or unacceptable) is correct.

### Laboratory tests

**Figure 5:**
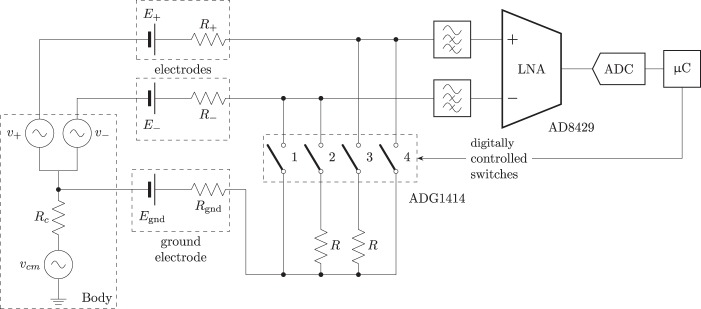
Schematic of the prototype test bench tested.

In the following phase, an experimental setup was arranged in order to check the validity of the methodology proposed with real electric signals similar to the ones found in real application; for this reason, a system as depicted in Fig. 5 was used. In this circuit, we use a classical configuration of common-mode plus differential signal to simulate the measured waveforms; the measurements are most commonly done using a differential configuration (all the switches open in Fig. 5) to be able to reject the common-mode signal.

A two-channel RIGOL-DG1022 waveform generator is utilized to simulate the input signal of an IONM acquisition system, including ${v}_{+}$, ${v}_{-}$ and the common mode voltage ${v}_{cm}$. These voltages are generated and combined with the help of a summing amplifier and a differential amplifier arrangement (not shown in the schematic), in order to obtain the desired differential and common mode value at each input. A series resistance is connected to the positive and negative electrodes in order to simulate the tissue-electrode contact impedance, following the simplifications presented in Section 2. Notice that the ground electrode is normally connected to the reference terminal of the instrumentation amplifier, either directly or through a voltage-follower setup. The ground electrode impedance is considered distributed into ${R}_{+}$ and ${R}_{-}$.

As indicated above, the differential voltage between the two electrodes is obtained by means of an AD8429 LNA. The analog output of the LNA is converted by means of a 16 bits digital to analog converter (ADC). Finally, the digital signal is registered by a microcontroller that is in charge of recording the signals and controlling the digital switches that configure the different connections required by the algorithm. For this purpose, an ADG1414 SPI-controlled array of switches is connected in parallel to the terminal inputs of the amplifier, in such a way that closing the corresponding switch allows either the parallel connection of the known resistance $R$ (switches 2, 3 in Fig. 5) or grounding (switches 1, 4 in Fig. 5) independently for each input.

The circuit utilized in the experiments was configured with the following features (voltages are peak-to-peak values):

- ${v}_{cm}=1\,{\rm{m}}{\rm{V}}$, sinusoidal at 50 Hz.
- ${v}_{+}=-\,{v}_{-}=15\,{\mu}{\rm{V}}$, from a previously recorded and digitalized neurological signal.
- ${R}_{c}$ is considered negligible. Given the high CMRR of the amplifier and the floating ground arrangement, it should not influence greatly the measurement.
- $$R=5\,{\rm{k}}\Omega $$.
- ${R}_{+},{R}_{-}$ varied among {100 Ω, 200 Ω, 500 Ω, 1 kΩ, 2.4 kΩ, 5 kΩ, 10 kΩ, 20 kΩ, 51 kΩ}.
- $$G=50{\rm{V}}/{\rm{V}}$$.
- The contact potential ${E}_{+}$, ${E}_{-}$, ${E}_{{\rm{gnd}}}$ were included in the voltage generated by the waveform generator.

The experiments were carried out with two different configurations of the amplifier: monopolar (where just one channel of the amplifier is used) and bipolar (with the amplifier connected in full differential mode). In the following, monopolar and bipolar refers to the amplifier configuration, and not on the type of pulse applied to ${v}_{+}$ or ${v}_{-}$; the latter is obtained from real-life registered signals that are reproduced by the arbitrary generators. The system itself does not apply any voltage, barring the unavoidable noise; bias current voltages leakage from the amplifier are cut by the high-pass filter capacitors. In the system we tested, the maximum amplitude that the system is able to accept is ±32 mV, but this is just related to the front-end used and the method can be applied with other, different designs.

**Figure 6:**
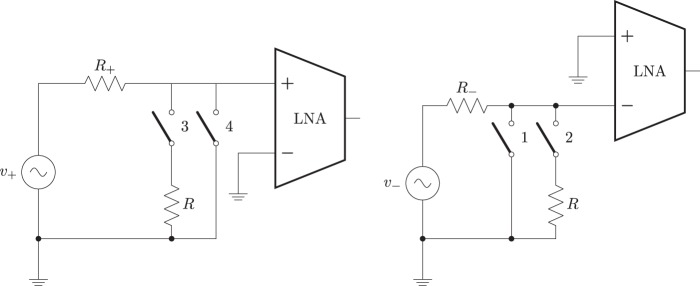
Schematic connection when measuring $\alpha$ (left) and $\beta$ (right) in monopolar configuration.

In the first set of tests, the value of $\alpha$ and $\beta$ were calculated by means of monopolar measurements. For this, one terminal input is grounded directly (without the help of the digital switch), while the opposite terminal receives the signal from the generator while the parallel load is switched. This configuration is depicted in Fig. 6.

From the voltages recorded during these experiments, (9) and (11) are applied to obtain the values of $\alpha$ and $\beta$.

**Figure 7:**
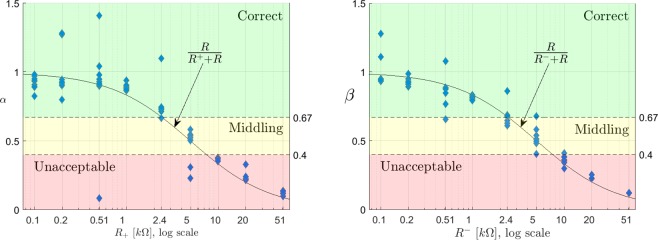
Value of $\alpha$ (left) and $\beta$ (right), obtained respectively with the inverting and non-inverting input grounded. Measured vs. calculated.

Figure 7 contain the results obtained after 10 measurements for different values of ${R}_{+}$ and ${R}_{-}$. The black line represents the theoretical value of $\alpha$ and $\beta$, calculated following (8) and provided that ${R}_{+}$ and ${R}_{-}$ are known. Consistency between experimental and theoretical results can be observed.

**Figure 8:**
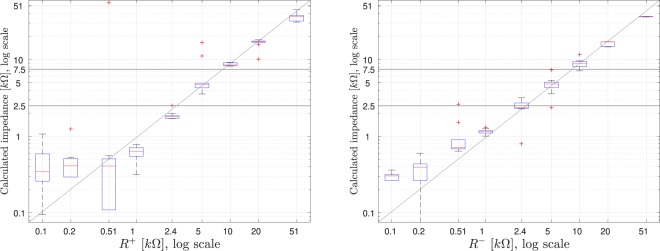
Boxplot of ${R}^{+}$ (left) and ${R}^{-}$ (right) calculated (ordinate), vs. resistor value located for the tests (abscissa).

Afterward ${R}_{+}$ and ${R}_{-}$ can be calculated once that $\alpha$ and $\beta$ are obtained, by way of Eq. (12). The results are shown in Fig. 8. The similarity between the resistor located for the tests and the calculated impedance can be observed. The thresholds for 2.5 kΩ and 7.5 kΩ are also shown in the figures.

Secondly, we carried out the assessment of both impedances in a bipolar and sequential fashion. In this case, no terminal is grounded and both electrodes are connected to the emulated biopotential sources. The digitally controlled switch is activated in order to obtain ${v}_{b}$, ${v}_{a}$, ${v}_{c}$ and ${v}_{d}$, so that the value of $\alpha$ and $\beta$ are computed in this order. A total of nine cases have been tested, resulting from the combination of three different resistor values for each terminal. Three significantly different impedance values have been utilized: 100 Ω, 5 kΩ and 51 kΩ.

**Figure 9:**
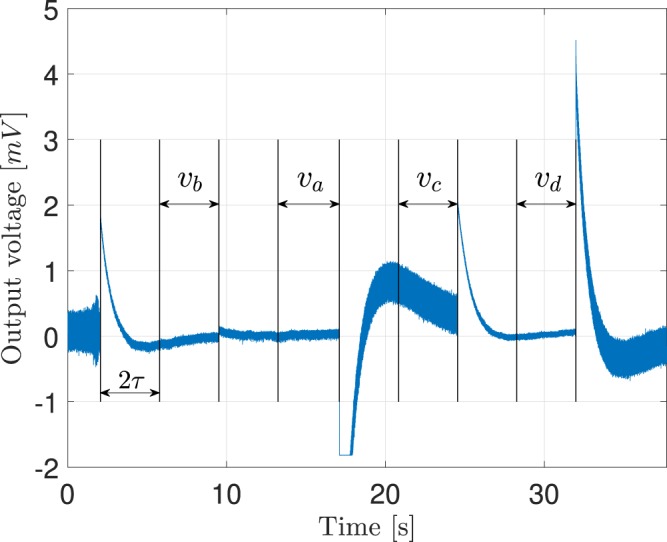
Gain corrected output voltage of one realization. Variation depending on the switch configuration.

Figure 9 shows the output voltage (without filtering) of the LNA for a realization of one of the experiments, where ${R}_{+}=100\,\Omega$ and ${R}_{-}=51\,{\rm{k}}\Omega$. During the contact impedance evaluation, the switches are toggled to measure the voltages for the four configurations. In the figure, the variation of the signal amplitude and the transient phenomenon due to the high pass filtering is clearly visible. The period $2\tau$, also indicated, is the chosen compromise between the steady-state condition and the overall speed of the measurement.

The realization of these experiments in a real device implies an additional challenge: the calculation of the RMS in real time. Computation of the RMS of each period requires a cumulative sum of the square of the samples obtained, followed by a division by the number of samples in the interval and the square root of the resulting value. This process has to be repeated four times, in order to compute all the values stated in (9). Because of its simplicity, the algorithm proposed can be implemented in a conventional 32-bits microcontroller. Following this procedure, the values obtained in the case shown in Fig. 9 are $\alpha =0.987$ and $\beta =0.124$.

**Figure 10:**
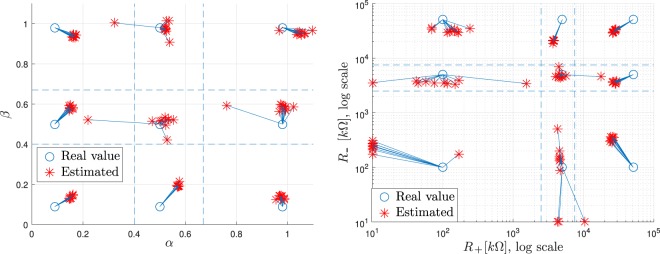
In the left side, value of $\alpha$ and $\beta$ coefficients obtained with sequential experiments. On the right side, the same results mapped to the resulting values of contact resistance.

In order to establish an analogy with the figures containing the results for the mathematical model and the simulation, the values obtained for $\alpha$ and $\beta$ have been arranged in Fig. 10 (left-side diagram). Note that the thresholds are now placed in coefficient values 0.4 (corresponding to $R=7.5\,{\rm{k}}\Omega$) and 0.67 ($R=2.5\,{\rm{k}}\Omega$).

From these results, it can be seen that the estimation error has increased with respect to the mathematical calculations and simulation, and moreover, there are two cases where the value of $\alpha$ overpasses the threshold separating the nine scenarios. In both cases, a middling value of impedance was assessed as bad, resulting in a false negative. These discrepancies are probably due to the effect of impulsive noise that may affect only to one part of the measurement, distorting the calculations.

The right-side diagram of Fig. 10 summarizes the results obtained for the calculation of the impedances and compares them with the theoretical calculations. For simplification and ease of representation, coefficient values larger than 1, which would indicate impossible negative contact resistances have been equated to 10 Ω. In a real implementation, these values will be considered as an equivalent input impedance of 0 Ω.

Considering the thresholds that divide the working cases of the system into the nine possible scenarios, a few realizations are classified as false negatives, corresponding to the cases where ${R}_{+}$, ${R}_{-}=5\,{\rm{k}}\Omega$, which is the threshold set between middling or unacceptable impedance, leading to a conservative algorithm. In a practical application, the three possible states of the impedance for each input terminal are indicated by a multicolour LED (red to mean a defective connection, yellow to indicate a connection in the limit of validity, and green for a good one). The false negatives are a minor defect and will lead to a recalculation of the impedance or, in the worst case, to a reconnection of the electrode.

### *In-vivo* tests

A series of tests has been performed on human volunteers (several of the authors) in the laboratories of the IIT and of the University Hospital “La Princesa”, in Madrid, respecting the internal protocols of the Institute; the experimental protocols were approved by the Ethical Committee of Clinical Research of Hospital de la Princesa (number of register PI-843, approved Dec. 21, 2015). The test has been duly authorized, and performed in accordance with the approved protocols, on two of the authors of the paper, whose have been informed and agreed to conduct the experiments, providing specific informed consent, and being fully aware of the risks and methodology of the experiment.

The experimental prototype used was battery-powered (with d.c. voltage never in excess of 10 V, and power dissipation well under 10 W), completely floating so that there were no possible leakage current towards main, and passive, not injecting any current. The experiment consisted on contact impedance measurements with the standard hospital equipment and the prototype system.

The contact impedances for four channels where previously measured by the standard IONM apparatus of the hospital, measuring values between 0.6 kΩ and 1.8 kΩ using the classical injected current methodology. Immediately after, the standard device was disconnected and the experimental device^23^ implementing the new method was connected, and the contact resistances assessed in five consecutive trials.

**Figure 11:**
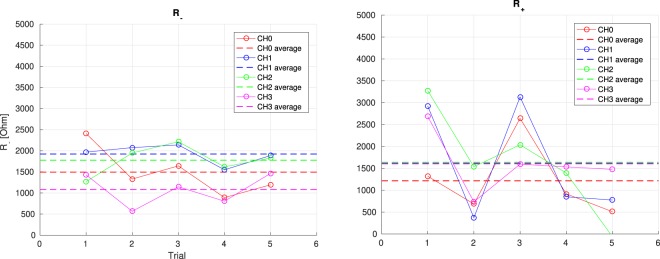
Result of the *in-vivo* measurements.

Figure 11 report the obtained results. As it can be seen, apart for the expected noisy nature of each of the consecutive trial, the averaged value is well in accordance with the measurement performed by the traditional instrument and, more important, is assessing the same scenario of “good” contact impedances.

## Discussion

A novel methodology for estimating the skin-electrode contact impedance is presented in this paper. This technique is based in the variation experienced by the measured voltage caused by the controlled connection of a parallel resistor to the input stage of the measurement device. Thus, injection of current to the patient is avoided and, by contrast, the signal utilized to carry out the assessment is the same random signal recorded from the patient. Problems such as skin abrasion, power consumption and complexity of the certification process are then reduced.

The capabilities of the new method have been evaluated and assessed by means of a mathematical model, a simulation, a prototype test bench analysis and several *in vivo* measurements. In the last two cases, the data is registered by a microcontroller that computes the algorithm proposed and gives back an estimation of the contact impedance of both positive and negative electrodes. It has been proved that computing the root mean square value of the output voltage and comparing the different measurements obtained allows the system to identify the correctness of the contact impedance for each electrode. This permits warning the user whether the measurements taken can be considered acceptable, doubtful or unacceptable.

The proposed method is found to be an improvement in current IONM technologies, and may imply a significant gain, particularly in battery powered autonomous systems, by omitting the circuitry needed to inject the probe current. The simplification of the certification procedure represents a substantial cost-cutting feature with respect to the same procedure for a device injecting current into the patient.

This method has been implemented in a wireless IONM system (WIONM) that is currently under field validation. The design has been patented^23^ and is currently in certification phase.

## Data Availability

Data has been gathered by experimental measurements directly on the several prototypes of the system. The author will share all the data available upon request.
